# Development and testing of a past year measure of sedentary behavior: the SIT-Q

**DOI:** 10.1186/1471-2458-14-899

**Published:** 2014-09-01

**Authors:** Brigid M Lynch, Christine M Friedenreich, Farah Khandwala, Andrew Liu, Joshua Nicholas, Ilona Csizmadi

**Affiliations:** Physical Activity Laboratory, Baker IDI Heart and Diabetes Institute, Melbourne, Australia; Melbourne School of Population and Global Health, Faculty of Medicine, Dentistry and Health Sciences, The University of Melbourne, Melbourne, Australia; Department of Population Health Research, Alberta Health Services - CancerControl Alberta, Calgary, Canada; Department of Oncology, Faculty of Medicine, University of Calgary, Calgary, Canada

**Keywords:** Sedentary behavior, Sitting time, Questionnaire, Cognitive interviewing, Reliability, Validity

## Abstract

**Background:**

Most sedentary behavior measures focus on occupational or leisure-time sitting. Our aim was to develop a comprehensive measure of adult sedentary behavior and establish its measurement properties.

**Method:**

The SIT-Q was developed through expert review (n = 7), cognitive interviewing (n = 11) and pilot testing (n = 34). A convenience sample of 82 adults from Calgary, Alberta, Canada, participated in the measurement property study. Test-retest reliability was assessed by intraclass correlation coefficients (ICCs) comparing two administrations of the SIT-Q conducted one month apart. Convergent validity was established using Spearman’s rho, by comparing the SIT-Q estimates of sedentary behaviour with values derived from a 7-Day Activity Diary.

**Results:**

The SIT-Q exhibited good face validity and acceptability during pilot testing. Within the measurement property study, the ICCs for test-retest reliability ranged from 0.31 for leisure-time computer use to 0.86 for occupational sitting. Total daily sitting demonstrated substantial correlation (ICC = 0.65, 95% CI: 0.49, 0.78). In terms of convergent validity, correlations varied from 0.19 for sitting during meals to 0.76 for occupational sitting. For total daily sitting, estimates derived from the SIT-Q and 7 Day Activity Diaries were moderately correlated (ρ = 0.53, p < 0.01).

**Conclusion:**

The SIT-Q has acceptable measurement properties for use in epidemiologic studies.

**Electronic supplementary material:**

The online version of this article (doi:10.1186/1471-2458-14-899) contains supplementary material, which is available to authorized users.

## Background

A growing body of evidence suggests that sedentary behavior (time spent sitting or reclining) is independently associated with all-cause and cardiovascular mortality [1–4], cardiovascular disease [5, 6], type 2 diabetes [7, 8], and some cancers [9]. Sedentary behavior appears to have physiological consequences, distinct from the effects associated with an absence of moderate- to vigorous-intensity physical activity, that may further contribute to chronic disease risk [10, 11].

Assessment of habitual patterns of physical activity and sedentary behavior are necessary to study associations with health outcomes [12, 13]. Measurement of physical activity and sedentary behavior by devices such as accelerometers or heart rate monitors can objectively measure duration, intensity and frequency. However, objective measures generally collect data over brief periods of time necessitating serial measurements for the ascertainment of habitual patterns of behavior. Further, objective methods do not provide behavioral context, and they may not be feasible for use with large, geographically dispersed samples. The cost of monitors, complexity of data processing and analysis, problems with compliance and burden on participants also limit their use in large population-based studies [14]. Hence, self-report methods of data collection are likely to remain the primary methods whereby activity will be quantified in epidemiological studies, especially for studies with limited economic resources [15, 16].

Existing self-report measures of sedentary behavior typically focus on either occupational sitting, or leisure-time pursuits such as television viewing and reading [17–21]. Additionally, some physical activity questionnaires, notably the International Physical Activity Questionnaire (IPAQ), include a global item that asks respondents to report their total sitting time [22]. Currently, few questionnaires attempt to assess adult sedentary behavior across multiple domains [23]. Such measures are necessary in order to determine whether the behavioral context is important in the associations between sedentary behavior and chronic disease [11, 24]. To address this need we developed the SIT-Q, a measure of habitual sedentary behaviors across occupation, transportation, household and leisure-time domains. Our aim was to develop a feasible and cost-effective measure of usual sedentary behavior for use in population cohort studies.

This report describes the development of the SIT-Q and the measurement properties of the questionnaire, specifically the test-retest reliability and convergent validity.

## Methods

**Figure 1:**
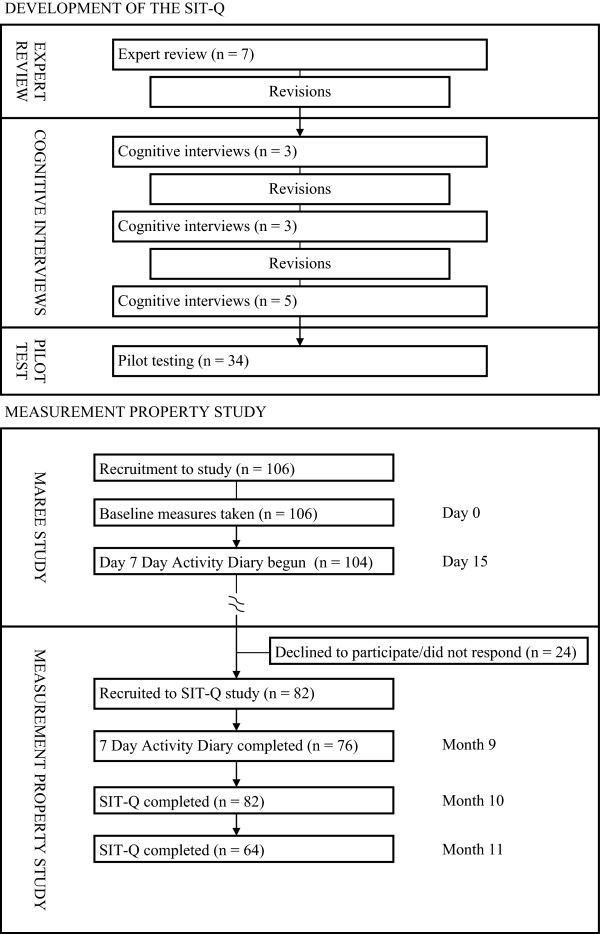
**Data collection protocol.**

This study was comprised of two distinct components: the development of the SIT-Q through a three staged approach of expert review, cognitive interviewing and pilot testing; and establishing the measurement properties of the SIT-Q (see Figure 1). The Alberta Cancer Research Ethics Committee of Alberta Health Services approved the procedures for all elements of this study.

### Development of the SIT-Q

The first version of the SIT-Q comprised 18 items across six different domains (sleeping and napping; transportation; employment and volunteer activities; meals; leisure time; household chores and do-it-yourself tasks). Common sitting or reclining tasks were identified from the Compendium of Physical Activities [25], the American Time Use Survey [26], and from a self-report measure of activity energy expenditure (the Sedentary Time and Activity Reporting Questionnaire; STAR-Q) developed by one of our study team [27]. We also created items pertaining to whether or not more prolonged sedentary behaviors were interrupted by standing or walking breaks, as patterns of sedentary behavioral accumulation have been shown to affect health outcomes [28, 29]. The SIT-Q recall period was past 12 months, and participants were instructed to estimate their usual activity pattern. Sedentary behaviors were assessed separately for weekdays and weekends, to facilitate recall.

#### Expert review

The SIT-Q was reviewed for logical and face validity by internationally recognized experts in sedentary behavior conceptualization, measurement, analysis and interpretation. Nine experts were identified and sent an email requesting their participation in the expert review; all agreed to participate and were sent the questionnaire. Experts were asked to provide general feedback and to specifically comment on: (i) the domains covered; (ii) whether the order items was logical and likely to help minimize double-counting of time; (iii) the clarity of instructions; and (iv) whether the response formats were appropriate for the data being collected.

#### Cognitive interviewing

Cognitive interviewing is an important, but often overlooked, step in questionnaire development [30, 31]. This method can identify misunderstandings of text that could lead to response error and can provide insight into ways in which respondents comprehend, retrieve and formulate their answer [31]. The two main methods used in cognitive interviewing are *think aloud* and *verbal probing*. When using the *think aloud* technique, the interviewee is asked to answer a question and additionally to talk about what he or she is thinking while reaching that answer. *Verbal probing* is used by the interviewer to probe for additional information relevant to the specific answer given [31].

Cognitive interviews were conducted by an MSc trained nurse-researcher to evaluate the feasibility of the SIT-Q as a self-administered questionnaire. Participants were primarily drawn from members of the Alberta Tomorrow Project (a province-wide longitudinal study) cohort, who had volunteered, but been deemed ineligible for, the Measuring Activity Related Energy Expenditure (MAREE) study. Convenience sampling was used to ascertain additional participants. All participants provided informed, signed consent prior to being interviewed.

The cognitive interviews were audio-taped and transcribed for analytic purposes. Based on these notes, the interviewer identified key themes representing sources of difficulty or confusion after each interview. These were summarized and discussed with the investigators after a “round” of three interviews. The insights gained from the interviews were used to revise the design of the questionnaire and to re-word the text for another round of cognitive interviewing.

#### Pilot testing

Convenience sampling was used to recruit participants for the pilot study. Forty-five volunteers were sent a copy of the SIT-Q (see Additional file 1), a consent form, a participant characteristics questionnaire, and a pilot testing feedback questionnaire. The pilot testing feedback questionnaire was designed to elicit information on the ease of the completion of the SIT-Q and on the clarity of the instructions and item wording. All questionnaires were completed by the participant in their own time and returned in a provided postage-paid envelope.

### Measurement property study

Participants of the MAREE Study (n = 106) were invited to participate in the SIT-Q measurement property study. The primary aim of the MAREE Study was to validate a self-report measure of activity energy expenditure (STAR-Q) against doubly-labeled water. As part of the MAREE Study protocol, participants completed a 7-Day Activity Diary two weeks after their recruitment (see Figure 1). The SIT-Q measurement property study entailed a second administration of the 7-Day Activity Diary, followed by two administrations of the SIT-Q, one month apart (Figure 1). The two administrations of the 7-Day Activity Diary allowed us to generate mean estimates of sedentary behaviors from diaries completed at different times of the year, but within the same reference period that was assessed by the SIT-Q.

#### 7-Day Activity Diary

The 7-Day Activity Diary involved recording the following details for each daily task: time task started; brief description of the task; position (recline, sit, stand, walk, in motion); activity group (self-care, household, occupation, walking for pleasure, care giving, transportation, yard work, exercise and conditioning, light leisure activity, sleeping, other); and physical effort (mainly sitting, mainly standing/no increase in heart rate, slight increase in heart rate, substantial increase in heart rate). The 7-day activity diary was adapted from Conway *et al*. [32] and was designed to ascertain sleep time as well as all activities (10 minutes or longer, or activities that caused a noticeable change in physical effort) and posture while awake. Participants were instructed to detail their activities in the 7-Day Activity Diary throughout the course of each day, rather than completing the diary each evening or at the end of the week.

Time spent in sedentary behaviors was estimated by two methods. The first estimated sedentary time from all tasks performed in a reclining or sitting position (postural definition). The second method assigned a metabolic equivalent (MET) level from the Compendium of Physical Activities [25] based on the brief description of the task and physical effort indicated, and was operationalized as behaviors ≤ 1.5 METs (MET definition). Sedentary behaviors were summarized into domains that matched those of the SIT-Q: meals; transportation; work, study and volunteering; childcare and eldercare; and, leisure time. Several of the activity group categories of the 7-Day Activity Diary were directly comparable to SIT-Q domains (transportation, occupation, care giving, and light leisure activity). We were not able to determine television viewing and computer use time separately from the 7-Day Activity Diary data (these were often jointly reported and hence amalgamated into leisure time sedentary behaviour). The self-care activity grouping corresponded with the meals domain (although self-care also included a minimal amount of time spent sitting during personal grooming activities), and a small amount of sitting time recorded within the household activity group was re-allocated to light leisure activity. There were no sedentary behaviors recorded within the walking for pleasure, yardwork or exercise and conditioning activity groups, and sleeping was excluded from our analyses as this is not considered a sedentary behavior.

#### SIT-Q

Participants were sent a copy of the SIT-Q one month after the 7-Day Activity Diary, and a second copy of the SIT-Q one month after the first (see Figure 1 for data collection overview; see Additional file 1 for SIT-Q). All questionnaires were returned by mail, and participants received up to three telephone calls to follow-up unreturned questionnaires. Importantly, all measures were completed within a 12-month period, commensurate with the 12-month recall period of the SIT-Q.

Data were manually entered into a database and any outlying responses were checked against the original questionnaires. Sedentary behaviors were assessed separately for weekdays and weekends within each domain except work, study and volunteering. Sedentary behavior during work, study and volunteering was reported based on weeks per year, days per week and hours per day. Given that most individuals did not work on a daily basis, the descriptive statistics for each type of “job” (work, study or volunteering) were summarized as hours or minutes of sedentary behavior in this domain per week. To facilitate estimation of total sedentary time, average minutes per day were calculated for each sedentary behavior.

#### Statistical analyses

Analyses were conducted using StataSE 12.0 (StataCorp LP, College Station, TX) and SAS/STAT® software (version 9.2) SAS System for PC. None of the sedentary behavior variables were normally distributed, except for total sedentary time. We therefore summarized sedentary behavior variables as median (interquartile range median; IQR) for all variables except for total sitting time, which was summarized as mean (standard deviation).

Test-retest reliability was assessed by intraclass correlation coefficients (ICCs) comparing two administrations of the SIT-Q, conducted one month apart. The ICCs for meals, transportation, work (incorporating study and volunteering), caring duties and leisure-time domains were estimated overall and separately for weekday and weekend days. We also examined the ICCs for television viewing and computer time, as these behaviours have frequently been assessed in sedentary behaviour measurement studies. ICCs were interpreted as follows: <0.40 indicated poor agreement; 0.40–0.74 fair to good agreement; ≥0.75 excellent agreement [21, 33]. Mean differences and 95% confidence intervals (CIs) between the first and second administrations of the SIT-Q were estimated. A Bland-Altman plot [34] of differences against means for each domain of sedentary behaviour was used to describe agreement at the individual level. Finally, the consistency with which participants rated how frequently they interrupted their prolonged sitting was assessed by percent agreement (%) and a weighted kappa statistic (wκ) [35] with the default weights matrix in Stata 12.0. Landis and Koch’s guide for interpreting agreement for categorical data was utilised: ≤0.20 slight; 0.21–0.40 fair; 0.41–0.60 moderate; 0.61-0.80 substantial; >0.80 almost perfect [36]. Statistical significance was set at P < 0.05.

Convergent validity was established using Spearman’s rank correlation coefficient (ρ), by comparing domain-specific estimates of sedentary behaviour from the first administration of the SIT-Q with the mean values derived from two administrations of the 7-Day Activity Diary, completed eight months apart. Comparisons were made across the activity group categories of the 7-Day Activity Diaries that corresponded closely with domains of the SIT-Q, namely self-care (this section included time sitting for meals and grooming); transportation; occupation; care giving; and light leisure activity. All sedentary behaviors were incorporated into these domains, and each domain was considered overall and separately for weekdays and weekend days. The strength of the correlations between the SIT-Q and the 7-Day Activity Diary was interpreted using the following: weak (<0.30); low (0.30–0.49); moderate (0.50–0.69); strong (0.70–0.89); very strong (≥0.90) [37]. Mean differences and 95% confidence intervals (CIs) between the SIT-Q and 7-Day Activity Diary estimates of sedentary behaviour were also estimated.

## Results

### Development of the SITQ

#### Expert review

The initial version of the SIT-Q contained 20 items grouped within six domains: sleeping and napping; transportation; employment and volunteer activities; meals; leisure-time; and, household chores and do-it-yourself. Formal review was completed by seven of the nine invited sedentary behavior experts. Overall, the feedback received was positive and few substantial changes were suggested.

Experts had been asked to respond specifically to four points. With regard to the *domains covered* by the SIT-Q, there was broad agreement that the questionnaire had covered the major domains. In relation to the *order of the SIT-Q items*, the expert reviewers all agreed that a logical flow is important for minimizing double-counting of time, and they concurred that the order of the SIT-Q items was sensible. One expert made the recommendation that a table of contents be included inside the front cover of the questionnaire: providing participants with an understanding of where and when they can report time spent in various seated tasks might also help to prevent some double-counting of time. Two of the reviewers recommended including driving to and from work in the transportation section, rather than within the Employment and Volunteer Activities domain, to prevent respondents from reporting this travel time twice. While most of the reviewers indicated that the *clarity of instructions* was good, two suggested that some of the instructions may be too lengthy, encouraging respondents to skim over or not read them at all. The difficulty in striking a balance between being comprehensive and parsimonious was acknowledged, and the two reviewers concluded that many of these issues would be resolved through the cognitive interview phase. A suggestion was made regarding the *response format* of the SIT-Q that, for some items, it would be helpful to provide options of “hours per day” in addition to “hours per week” because some participants might not engage in this behavior on a daily basis.

A number of additional recommendations arose from the expert review, including a suggestion to add an item on snacking behavior while watching television. The expert who suggested this inclusion thought it would provide useful insight for disentangling the contributions of television time and high energy snacks to outcomes such as adiposity and metabolic dysfunction. Refinements based on the suggestions provided by expert reviewers were made to the SIT-Q accordingly.

#### Cognitive interviewing

Eleven cognitive interviews were conducted, each one lasting between one and a half and two hours. Three males (mean age = 51.0 years, SD = 13.9 years) and eight females (mean age = 44.9 years, SD = 7.5 years) participated. Further details on the characteristics of participants in the cognitive interviews and pilot testing can be found in Additional file 2. Three rounds of testing were conducted until, during the final round, five successive interviews did not identify any new sources of misunderstanding.

Table 1 outlines key sources of difficulty or confusion identified by the cognitive interviews, and the resultant changes made to the SIT-Q. In the final round of interviews, all changes that had been made due to reported issues in rounds 1 and 2 were well received and appeared to make comprehension easier for participants.

**Table 1:** **Sources of misunderstanding or difficulty identified during the cognitive interviews (n = 11)**

Sources of misunderstanding/confusion	Initial wording of SIT-Q	Revised wording of SIT-Q
***Instructions***		
• Confusion about use of ‘activity’ to describe time spent sitting or lying down.	• Do your best to estimate your usual activity pattern.	• The amount of time you spent sitting or lying down may have varied over the past 12 months. Do your best to estimate your usual pattern over the past 12 months.
• Uncertainty about the concept of ‘double-counting’.	• Do not double-count time. For each of the activities only count the time where this was your main activity. The total hours should not add up to more than 24 hours.	• For each of the sitting tasks only count the time where this was your main focus. For example, if you spent one hour sitting on the sofa reading a book while you had a CD on in the background, count this time as one hour reading (do not also ‘double count’ as one hour listening to music).
***Sleeping and Napping***		
• Uncertainty whether to include time spent lying in bed before falling asleep as sleep time.	• Please record the usual number of hours of sleep for weekdays and weekends.	• Please record how long you usually slept on weekdays and weekends. This may include time you spent lying quietly while waiting to fall asleep, or after awakening.
• Understanding what was meant by ‘usually take a daily nap’ was difficult for some.	• If you did not usually take a daily nap on weekdays or weekends over the past 12 months, please write “0” in the response section. How long did you usually nap per day?	• Did you take a nap each day, on either weekdays or weekends, over the past 12 months? How long did you usually nap per day (do not include occasional naps)?
***Transportation***		
• Difficulty incorporating semi-regular driving that is neither usual daily trips nor a holiday.	• Please record the usual amount of time you spent sitting during transportation over the past 12 months. Do not include one-off trips like holidays. Record the usual hours per day for weekdays and weekends.	Note: no change was made in this instance; response will be dependent on participant’s perception of “usual”.
• Participants indicated that separate questions for driving a car and being a passenger were not necessary.	• Record the usual hours per day for weekdays and weekends (a) driving a car (b) sitting as a passenger in a car, bus, train etc.	• How long did you usually spend sitting during transport per day?
• Some misunderstanding about instruction regarding ‘transportation while at work’.	• Do not include time sitting in an automobile while at work.	• Do not report time spent sitting during transportation as part of your job (you will be asked about this later).
***Work, study and volunteering***		
• One participant recorded 52 weeks spent in her job, even though she verbally said she only worked 48 weeks, with four weeks vacation.	• Weeks per year? Note: no instruction other than a heading for the response section	• Do not include holiday time here, even if it is paid vacation.
		• How many weeks in the past 12 months did you do job # 1?
• Difficulty in separating out times spent doing light office tasks like typing or reading, as these were done at the same time as talking to others. Participants reported multi-tasking was frequent, and hence it would be easier to provide an overall estimate of sitting time at work, rather than for separate work tasks.	• Record the amount of time you spent doing the following sitting tasks as part of your job over the past 12 months:	• How much time per day did you spend sitting for your job? (include driving and travelling while doing this job; do not include time commuting to and from this job).
	(a) driving or travelling in a car (do not include operating heavy machinery)	
	(b) non-strenuous tasks while sitting (typing, reading, light assembly)	
	(c) sitting while talking to others (on the telephone, during meetings).	
• Need for response option of “never” for the items about breaking up sedentary time.	• How often do you ‘break up’ the time you spend sitting in job # 1?	• How often did you ‘break up’ the time you spent sitting in job # 1?
	(a) less than hourly	(a) less than hourly
	(b) hourly	(b) hourly
	(c) half hourly	(c) half hourly
	(d) every 10 minutes	(d) every 10 minutes
	(e) every 5 minutes.	(d) every 5 minutes
		(f) I did not sit for more than 30 minutes in a day.
***Household chores and childcare***		
• Participants spent almost no time sitting for any household chores. All such tasks were done from a standing position. Participants could not identify with the example tasks provided.	• Chores done sitting down on weekdays and weekends (examples: food preparation, folding clean clothes, household accounts, polishing silver).	Note: household chores section removed from SIT-Q.
• Participants felt that the question and examples about childcare in this section could also apply for looking after elderly parents or other family members with disabilities.	• Child-care tasks while sitting down on weekdays and weekends (examples: nursing a baby, feeding a child, reading to a child).	• How long did you usually spend sitting or lying down while caring for your child per day? (examples: nursing baby, helping child with homework).
		• How long did you usually spend sitting down while caring for elderly family member per day? (examples: reading aloud, assistance with eating meals).

Other, more general, issues raised during the cognitive interviews included difficulty with the concept of ‘usual’ time, particularly if participants had a varied routine. For example, one participant, a health professional, worked four days per week on a hospital ward and one day per week in her office. Other participants had shared custody of their children. Their usual routine varied substantially depending on whether or not their children were staying with them. Seasonal variation also made it difficult for participants to define their usual sedentary behavior, particularly within the leisure-time domain. A number of participants indicated that they were more sedentary during the winter months than in summer.

The feedback received during the cognitive interviews regarding the breaks in sedentary time items was positive. Participants indicated that the questions were clearly stated and did not create any confusion. Feedback from participants also led to changes in the order of domains, so as to improve the logical flow of questioning from one topic to the next. The initial SIT-Q assessed by the cognitive interviews was ordered: (1) sleeping and napping; (2) transportation; (3) work, study and volunteering; (4) meals; (5) leisure-time; and, (6) household chores and do-it-yourself. The revised version of the SIT-Q (to be pilot tested) was ordered: (1) sleeping and napping; (2) meals; (3) transportation; (4) work, study and volunteering; (5) childcare and elder care; (6) light leisure and relaxing; and, (7) final questions. The revised SIT-Q incorporated ‘final questions’ , an additional section with open-ended responses to allow participants to record any additional sedentary behaviors not covered by the questionnaire.

#### Pilot testing

The pilot study phase was completed by 34 participants (76% response rate), of whom 14 were male (mean age = 38.0 years, SD = 19.5 years) and 20 were female (mean age = 44.1 years, SD = 10.8 years). The total time spent in sedentary behaviors (excluding sleeping and napping) was 12.0 hours per day (SD = 3.9), representing 73% of participants’ waking time. There was no significant difference between the total hours of sitting time for men (mean = 12.8, SD = 4.0) and women (mean = 11.5, SD = 3.9). Each SIT-Q returned was completed correctly; none of the responses were outside of expected parameters. Whilst there were no true missing data, approximately half of the pilot study sample simply left sections blank that did not apply to them, rather than entering “0” as instructed by the questionnaire. Participants rated the SIT-Q highly in terms of ease of use: on a scale of 1 (not at all easy) to 5 (very easy) the mean rating was 4.3, and 50% of the pilot study sample gave a rating of 5. There was considerable variation in the SIT-Q domains considered the easiest to complete: 36% reported sleeping and napping was the easiest section to complete, while 24% reported meals and 12% reported transportation. All participants indicated that their usual sedentary behavior was covered by the SIT-Q items, and no additional tasks were suggested for inclusion in the questionnaire.

### Measurement property study

Eighty-two (77%) participants enrolled in the MAREE Study agreed to take part in the SIT-Q measurement property study. Of these, 34 were male (mean age = 51.2 years, SD = 6.7 years; mean BMI = 25.6 kg/m^2^, SD = 3.2 kg/m^2^) and 47 were female (mean age = 45.9 years, SD = 8.6 years; mean BMI = 23.7 kg/m^2^, SD = 2.8 kg/m^2^). For further details on the characteristics of the sample, see Additional file 3. The second administration of the 7-Day Activity Diary was completed by 76 participants. Mean values of the summary variables were calculated from the two different administrations of the 7-Day Activity Diary; 74 complete records were available for analysis. All 82 participants completed the first administration of the SIT-Q; 64 completed the second administration of the SIT-Q.

**Figure 2:**
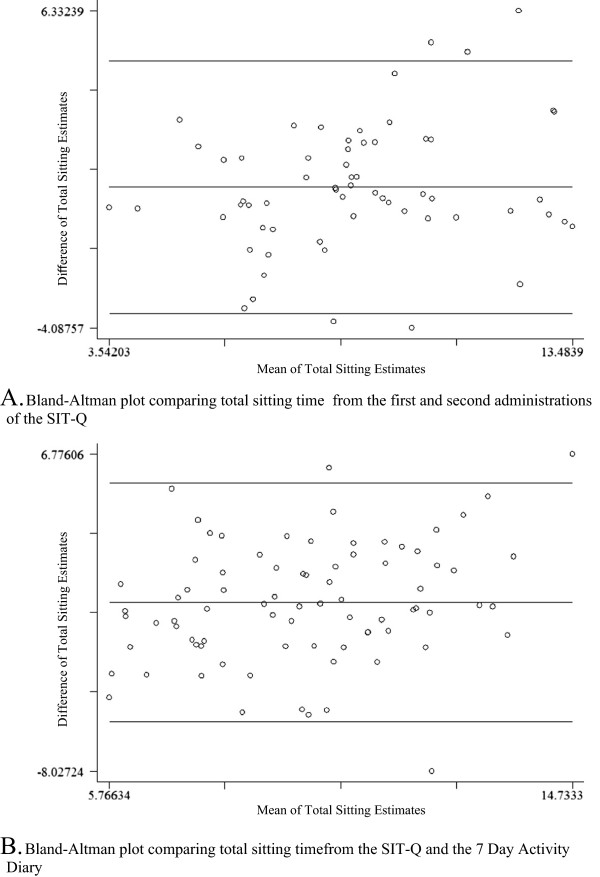
**Bland-Altman plots for test-retest reliability and convergent validity of the SIT-Q. A**. Bland-Altman plots of total sitting time (h/day) estimated by the SIT-Q during two administrations, one month apart, with means (solid lines) and limits of agreement (dashed lines). **B**. Bland-Altman plot of total sitting time (h/day) estimated by the first administration of the SIT-Q and the 7-Day Activity Diary.

Overall, there was a mean difference of 52 minutes between the first and second administrations of the SIT-Q. There was little mean difference noted across the meals, transportation or childcare and eldercare domains, however the second administration saw an overall decrease of approximately half an hour for the work, study and volunteering, and leisure time domains. In terms of test-retest reliability, the ICCs ranged from 0.31 (poor) for computer use during leisure time to 0.86 (excellent) for occupational sitting (see Table 2). Total daily sitting demonstrated fair to good correlation (ICC = 0.65, 95% CI: 0.49, 0.78). Figure 2 shows the Bland-Altman plot for total sitting time (h/day), with wide 95% limits of agreement (-3.62, 4.69). Overall, participants tended to report shorter periods of sedentary behaviour in the second administration of the SIT-Q. However the plot demonstrates that there was little systematic variability in reporting, although there were a few outliers indicating some instances of high variability at the upper end.

**Table 2:** **Results comparing sitting time (h/day) measured by two administrations of the SIT-Q, one month apart (n = 64)**

	SIT-Q	SIT-Q		
	First administration	Second administration	Mean difference (95% CI)	ICC (95% CI)
**Meals** (h/day)	1.07 (0.52)	1.00 (0.56)	0.06 (-0.03, 0.16)	0.60 (0.42, 0.74)
*weekday only*	1.00 (0.50)	1.00 (0.63)	0.03 (-0.06, 0.12)	0.65 (0.48, 0.77)
*weekend only*	1.25 (0.75)	1.00 (0.71)	0.14 (0, 0.29)	0.41 (0.18, 0.59)
**Transportation** (h/day)	0.82 (0.61)	0.83 (0.75)	-0.05 (-0.15, 0.06)	0.59 (0.41, 0.73)
*weekday only*	0.75 (0.50)	0.75 (0.50)	-0.04 (-0.14, 0.06)	0.65 (0.48, 0.77)
*weekend only*	1.00 (1.00)	1.00 (1.00)	-0.06 (-0.26, 0.13)	0.51 (0.30, 0.67)
**Work, study and volunteering** (h/day)	2.83 (4.22)	2.36 (4.42)	-0.25 (-0.54, 0.04)	0.86 (0.78, 0.91)
**Childcare and elder care** (h/day)	0 (0)	0 (0)	0.13 (0.01, 0.24)	0.59 (0.40, 0.73)
*weekday only*	0 (0)	0 (0)	0.12 (0.01, 0.24)	0.60 (0.41, 0.73)
*weekend only*	0 (0)	0 (0)	0.13 (-0.01, 0.28)	0.59 (0.40, 0.73)
**Television viewing time** (h/day)	1.73 (1.29)	1.50 (1.39)	0.12 (-0.03, 0.28)	0.84 (0.75, 0.90)
*weekday only*	1.50 (1.50)	1.50 (1.50)	0.07 (-0.11, 0.24)	0.82 (0.72, 0.89)
*weekend only*	2.00 (1.67)	2.00 (2.00)	0.27 (-0.01, 0.55)	0.69 (0.53, 0.80)
**Computer use at home** (h/day)	1.00 (1.00)	0.64 (0.64)	0.12 (-0.03, 0.28)	0.31 (0.07, 0.52)
*weekday only*	1.00 (1.00)	0.50 (0.50)	0.31 (0.06, 0.56)	0.25 (0, 0.47)
*weekend only*	1.00 (1.00)	0.50 (0.71)	0.23 (0.04, 0.43)	0.42 (0.19, 0.60)
**Leisure time** (h/day)	4.50 (2.69)	3.82 (2.39)	0.65 (0.29, 1.02)	0.61 (0.43, 0.74)
*weekday only*	3.71 (3.08)	3.00 (2.00)	0.58 (0.21, 0.95)	0.63 (0.45, 0.76)
*weekend only*	6.00 (3.50)	4.71 (3.42)	0.79 (0.21, 1.36)	0.51 (0.31, 0.68)
**Total sitting time** (h/day)	9.48 (2.82)	8.59 (2.36)	0.54 (0.01, 1.07)	0.65 (0.49, 0.78)

First and second administration results presented are median (IQR) for all variables except for total sitting time, which is presented as mean (standard deviation).

In response to the question *How often did you “break up” the time you spent sitting* during their primary “job” (response options: *I did not sit for more than 30 minutes in a day; less than hourly; half hourly, every 10 minutes; every 5 minutes*), participants’ absolute agreement between the two administrations of the SIT-Q was 55%, and the weighted kappa was 0.49. Similar results were obtained for the item pertaining to breaking up time spent sitting whilst watching television (percent agreement = 61%; wκ = 0.50) and for the item assessing how often participants ate snack foods whilst watching television (percent agreement = 56%; wκ = 0.48). Hence, the two items referring to breaks in sitting time, and the item referring to frequency of snack food consumption, demonstrated moderate agreement.

There were 69 participants with mean diary and first SIT-Q data. Overall, there was less than ten minutes difference between the SIT-Q and 7-Day Activity Diary estimates of total daily sedentary behavior. Compared with the 7-Day Activity Diary, the SIT-Q estimated over half an hour less within the meals and transportation domains. There was relatively little difference between the different methods of sedentary behavior assessment for the work, study and volunteering or childcare and eldercare domains. A substantial difference was noted within the leisure time domain; the SIT-Q estimated nearly two hours per day more leisure time sedentary behavior.

Correlations representing the convergent validity varied from 0.19 (weak) for sitting during meals to 0.76 (strong) for occupational sitting (see Table 3). For total daily sitting, estimates derived from the SIT-Q and 7- Day Activity Diaries were moderately correlated (ρ = 0.53, p < 0.01). There were only minor differences in the estimates of time spent in sedentary behaviours based on postural or MET-based definitions from the 7-Day Activity Diaries. The correlations were somewhat lower for sitting during meals (postural definition) in comparison to the MET-based definition; however the correlation coefficients were equivalent across other categories and for total sedentary time. The Bland-Altman plot for total sitting time (h/day) is shown in Figure 2. This plot also shows wide confidence intervals but little systematic variability in reporting.

**Table 3:** **Results comparing sitting time measured by the SIT-Q to estimates derived from Seven-Day Activity Diaries (both postural and MET-based definitions of sedentary) (n = 69)**

	Diary – postural definition			Diary – MET-based definition		
		Mean difference (95% CI)	Spearman’s ρ		Mean difference (95% CI)	Spearman’s ρ
**Meals** (h/day)	1.52 (0.79)	-0.58 (-0.75, -0.42)	0.19 (p = 0.11)	1.55 (0.74)	-0.65 (-0.83, -0.48)	0.29 (p = 0.01)
*weekday only*	1.47 (0.81)	-0.63 (-0.81, -0.44)	0.07 (p = 0.58)	1.47 (0.80)	-0.69 (-0.88, -0.50)	0.23 (p = 0.07)
*weekend only*	1.52 (1.03)	-0.45 (-0.66, -0.25)	0.36 (p < 0.01)	1.61 (0.95)	-0.52 (-0.75, -0.28)	0.37 (p < 0.01)
**Transportation** (h/day)	1.34 (0.91)	-0.53 (-0.67, -0.38)	0.37 (p < 0.01)	1.37 (0.92)	-0.54 (-0.69, -0.39)	0.34 (p < 0.01)
*weekday only*	1.21 (0.93)	-0.60 (-0.77, -0.43)	0.39 (p < 0.01)	1.27 (0.92)	-0.62 (-0.79, -0.45)	0.37 (p < 0.01)
*weekend only*	1.29 (1.27)	-0.38 (-0.64, -0.11)	0.11 (p = 0.38)	1.26 (1.27)	-0.38 (-0.64, -0.12	0.11 (p = 0.37)
**Work, Study and Volunteering** (h/day)	2.64 (3.48)	-0.17 (-0.51, 0.16)	0.76 (p < 0.01)	2.72 (3.30)	-0.24 (-0.59, 0.11)	0.75 (p < 0.01)
**Childcare and elder care** (h/day)	0 (0)	0.11 (-0.01, 0.23)	0.49 (p < 0.01)	0 (0.02)	0.15 (0.03, 0.27)	0.46 (p < 0.01)
*weekday only*	0 (0)	0.11 (-0.03, 0.24)	0.47 (p < 0.01)	0 (0.03)	0.16 (0.01, 0.29)	0.36 (p < 0.01)
*weekend only*	0 (0)	0.15 (-0.01, 0.30)	0.61 (p < 0.01)	0 (0)	0.18 (0.03, 0.34)	0.53 (p < 0.01)
**Leisure time** (h/day)	2.57 (2.01)	1.93 (1.38, 2.48)	0.26 (p = 0.03)	2.61 (2.12)	1.94 (1.39, 2.49)	0.26 (p = 0.03)
*weekday only*	2.33 (2.28)	1.68 (1.08, 2.29)	0.31 (p = 0.01)	2.33 (2.35)	1.68 (1.08, 2.28)	0.32 (p = 0.01)
*weekend only*	3.24 (2.89)	2.44 (1.68, 3.20)	0.14 (p = 0.26)	2.75 (3.50)	3.01 (2.17, 3.86)	0.09 (p = 0.45)
**Total sitting time** (h/day)	9.50 (2.36)	-0.15 (-0.78, 0.47)	0.53 (p < 0.01)	9.26 (2.25)	0.09 (-0.50, 0.68)	0.52 (p < 0.01)

## Discussion

We adopted a three-staged approach to the development and refinement of the SIT-Q: expert review, cognitive interviewing and pilot-testing. Broadly, our findings suggest that the SIT-Q exhibits good face validity and is highly acceptable to questionnaire respondents. Whilst helpful suggestions were provided through the process of expert review, it was encouraging that no substantial changes to the SIT-Q were recommended: this finding indicated consistency in the understanding of these behaviors between the authors and content experts. Findings from the cognitive interviews lead to significant restructuring of questions and changes in wording, as participants’ perception of the meaning of questions was sometimes quite different from what had been intended. Improvement in participant comprehension was achieved through revisions to the wording of instructions and questions. However, some factors such as seasonal differences and variable work or family routines remained limitations in the context of past 12-month recall. The pilot study confirmed that the SIT-Q was acceptable to respondents, and that most common sedentary behaviors were included in the questionnaire.

The second administration of the SIT-Q generated an estimate of total sedentary behavior that was 52 less than derived by the first administration. Whilst the difference between total sedentary behavior estimates from the first administration of the SIT-Q and the 7-Day Activity Diary were small, we noted substantial differences between methods in relation to leisure-time sedentary behavior. The first administration of the SIT-Q appears likely to have over-estimated leisure time sitting, possibly due to concurrent computer and television use. These two behaviors could not be disentangled from the diary data, as many participants reported these as occurring at the same time. We note that leisure-time sedentary behavior was over 40 minutes less in the second administration of the SIT-Q; perhaps familiarity with methods of recalling and reporting sitting time resulted in less of the ‘double counting’ that had been identified as an issue in the cognitive interviewing process.

The psychometric properties of the SIT-Q are comparable to the test-retest reliability and convergent validity established for other sedentary behavior questionnaires. The test-retest reliability for total sitting time (ICC = 0.65) was somewhat higher than the ICC presented for total sitting time by Gardiner *et al*. (0.52) [38], and somewhat lower than the ICCs reported by Salmon *et al*. (0.79) [20] and Rosenberg *et al*. (weekday = 0.85; weekend = 0.77) [19]. However, the estimates of total sitting time generated by other questionnaires did not encompass the variety of domains, or number of different sedentary behaviors covered by the SIT-Q. The SIT-Q performed favorably when measuring television viewing time (ICC = 0.84), compared to findings from Gardiner *et al*. (ICC = 0.76) [39], Marshall *et al*. (ICC for men, weekdays = 0.65; weekends = 0.62, Pearson’s *r* for women, weekdays = 0.79, weekends = 0.57) [18], Salmon *et al*. (ICC = 0.82) [20] and Rosenberg *et al*. (ICC weekdays = 0.86; weekends = 0.84) [19]. Only Marshall *et al*. reported the test-retest properties for occupational sitting (ICC for men, weekdays = 0.86, ICC for women, weekdays = 0.79); their results were similar to the SIT-Q results (ICC = 0.86) [18].

The SIT-Q demonstrated moderate validity overall. In terms of the validity of other sedentary behavior measures, only Salmon *et al*. [20] and Marshall *et al*. [18] compared their sedentary behavior questionnaires against activity logs or diaries, however neither of these studies determined the convergent validity for their measured estimate of total sitting time. The SIT-Q’s estimate of occupational sitting (ρ = 0.76) compared favorably to the item examined by Marshall *et al*. (*r* for men, weekdays = 0.74, for women, weekdays = 0.69) [18]. Sitting whilst driving in the Salmon *et al*. scale demonstrated low correlation (ρ = 0.30) compared to a 3-Day Log; [20] a similar correlation was found for sitting during transportation in the SIT-Q (ρ = 0.37).

A major strength of the SIT-Q is the rigorous methodological approach used to develop the instrument. The three-stage process of expert review, cognitive interviewing and pilot testing was critical for minimizing future participant reporting errors by ensuring that items and instructions were worded in a manner appropriate for the target population. However, it is important that the SIT-Q is used within the context for which it was designed. The SIT-Q was developed for use in epidemiological studies where habitual patterns of sedentary behavior are of interest in relation to chronic disease development and progression; it may not be a sensitive measure of change in sedentary behavior. Using the 7-Day Activity Diary as our criterion measure enabled us to estimate the validity of domain-specific estimates of sedentary behavior. Further, incorporating two administrations of the diaries and using the mean of these estimates likely reduced intra-individual variation in the criterion measure.

The study has a number of limitations that must be considered. The convenience samples drawn for both the cognitive interviews and pilot testing were comprised of individuals from Calgary, Canada, whose lifestyle may differ significantly from residents of other countries, particularly with regards to outdoor pursuits. Further, all study participants were urban residents, and hence the experiences of adults who live and work in rural areas were not incorporated into the development of the SIT-Q. Men and members of non-white ethnicities were under-represented, and study participants tended to be well educated and have sedentary, white-collar jobs. Whilst estimates of sedentary behavior derived from the 7-Day Activity Diaries may be more accurate than a seven day recall questionnaire because of careful daily accounting of time spent in various activities, they will still be subject to reporting errors and biases. In future work we will determine the criterion validity of the SIT-Q by comparing estimates of sedentary behavior against data collected using the activPAL® inclinometer (PAL Technologies, Glasgow, Scotland).

## Conclusion

The SIT-Q was developed to fill a need for a rigorously tested, comprehensive measure of habitual adult sedentary behavior. It is the first sedentary behavior questionnaire to be developed and refined using expert review, cognitive interviewing and pilot-testing. Overall, the SIT-Q demonstrated fair to good test-retest reliability and moderate validity. Items pertaining to behaviors that occur in more structured contexts, such as television viewing or occupational sitting, displayed the strongest psychometric properties [23]. These findings, coupled with the high acceptability of the questionnaire from respondents in the pilot study, suggest that our aim to develop a feasible measure of usual sedentary behavior for use in population cohort studies has been achieved. Given the emerging evidence suggesting that sedentary behavior is a modifiable lifestyle risk factor for numerous chronic diseases, the SIT-Q should be a useful addition to available methods of sedentary behavior assessment.

## Electronic supplementary material

Additional file 1: **SIT-Q: this is the version of the SIT-Q used in the measurement properties study.** (PDF 185 KB)

Additional file 2: **Sociodemographic characteristics of cognitive interview and pilot study participants.** (CSV 1 KB)

Additional file 3:: **Sociodemographic characteristics of measurement property study participants.** (CSV 867 bytes)
