# Supplementary material for: Development and testing of a past year measure of sedentary behavior: the SIT-Q
Source: BMC Public Health. 2014 Sep 1;14:899. doi: 10.1186/1471-2458-14-899 (PMC4158075; doi:10.1186/1471-2458-14-899)
Supplement: Supplementary file 1 — Additional file 1: SIT-Q: this is the version of the SIT-Q used in the measurement properties study. (PDF 185 KB) [file 12889_2013_7017_MOESM1_ESM.pdf]

SIT-Q

Instructions:

- These questions are about the usual amount of time over the past 12 months that you spent sitting or lying down.
- The amount of time you spent sitting or lying down may have varied over the past 12 months. Do your best to estimate your usual pattern over the past 12 months.
- If you did not participate in a particular sitting task, please write "0" in the time response field.
- For each of the sitting tasks only count the time where this was your main focus. For example, if you spent one hour sitting on the sofa reading a book while you had a CD on in the background, count this time as one hour reading (do not also 'double count' as one hour listening to music).

If you have any questions please contact
Telephone:
Email:

ID

The SIT-Q is organized into seven sections, each asking about sitting or lying down in different settings.

Section 1 – Sleeping and Napping……………………………………………….……….3

Section 2 – Meals ………………………………………………………………………4

Section 3 – Transportation ……………………..………………. ……………………5

Section 4 – Work, Study and Volunteering…………………………………………6

Section 5 – Childcare and Elder Care ……………………………………..…..........11

Section 6 – Light Leisure and Relaxing...…………………….………………..12

Section 7 – Final Questions ……………………………………………………..14

SECTION 1 – SLEEPING AND NAPPING

Sleeping and napping are an important part of your daily routine.

If you do shift work or you have variable sleeping patterns, please try to estimate the average number of hours in your sleep period, whether this is during the night or day.

SLEEPING

Think about how many hours you usually slept each night over the past 12 months.

Please record how long you usually slept on weekdays and weekends. This may include time you spent lying quietly while waiting to fall asleep, or after awakening.

1. How long did you usually sleep per night?<br>*(include time spent lying quietly while waiting to fall asleep, or after awakening)*	___hr ___min<br>*(weekday)*	___hr ___min<br>*(weekend)*

NAPPING

A nap is a brief sleep, often during the day. A nap can be taken in a chair as well as in a bed.

Did you take a nap each day, on either weekdays or weekends, over the past 12 months?

➔ If no, please write "0" in the response section, below.

2. How long did you usually nap per day?<br>*(do not include occasional naps)*	___hr ___min<br>*(weekday)*	___hr ___min<br>*(weekend)*

SECTION 2 - MEALS

Eating is a task we don't often think about, but it can take up quite a bit of time each day.

Please think about the amount of time you usually spent sitting for meals over the past 12 months:

- do report times when your main focus was eating, including eating out
- do report the amount of time you spent between sitting down and being finished with a meal (leaving the table)
- do not include time spent preparing food
- do not include times you were eating while doing other things, like snacking while watching TV (you will be asked about this later).

3. How long did you usually spend sitting for meals per day?	___hr ___min *(weekday)*	___hr ___min *(weekend)*

SECTION 3 – TRANSPORTATION

This section refers to the time you spent sitting during transportation (travelling in a car, bus, train, etc.) in the past 12 months:

- do report time spent as either a driver or a passenger
- do report time spent commuting to and from work
- do not report time spent sitting during transportation as part of your job (you will be asked about this later)
- do not include occasional travel such as holidays
- do not include transportation on motorcycles, scooters or bicycles.

4. How long did you usually spend sitting during transport per day?	___hr ___min<br>*(weekday)*	___hr ___min<br>*(weekend)*

SECTION 4 – WORK, STUDY AND VOLUNTEERING

"Work" refers to your occupation or your job - all tasks done to earn money or make a living. You may work full-time or part-time; you may work for a company or be self-employed.

"Study" refers to formal educational activities related to school, technical college or university.

"Volunteering" refers to work that you do for no pay, such as helping at a hospital, church or sports club.

Please complete one response section for each type of work, study or volunteering you did in the past 12 months:

- there is space to record up to four different types of work, study or volunteering you may have done over the past 12 months
- do include the usual amount of time that you spent sitting down as part of your work, study or volunteering
- do not record holiday time here, even if it is paid vacation.

➔ If you did not do any work, study or volunteering in the past 12 months, please skip to **Section 5** on page 11.

Choose type of “job”: ☐ work ☐ study ☐ volunteering

Please name Job #1: ______________________________

5a. How many weeks in the <u>past 12 months</u> did you do Job # 1? ___ weeks

6a. How many days <u>per week</u> did you do Job # 1? ___ days

7a. How much time <u>per day</u> did you spend <u>sitting</u> for Job # 1? ___hr ___min
(include driving and travelling while doing this job; do not include time commuting to and from this job)

Think about the total time you spent sitting during Job # 1. We are interested in how often you stood up and moved around to “break up” the time you spent sitting. For example, you might have taken short walks to get a drink of water, to collect a document from the printer or to talk to someone else in the office.

8a. How often did you “break up” the time you spent <u>sitting</u> in Job # 1?

☐ *(less than hourly)* ☐ *(hourly)* ☐ *(half hourly)* ☐ *(every 10 mins)* ☐ *(every 5 mins)*

OR ☐ I did not sit for more than 30 minutes in a day

➔ Did you have any other work, study or volunteering “jobs” in the past 12 months? If so, continue on the next page.

➔ If you did not have any other jobs, please skip to **Section 5** on page 11.

Choose type of "job": ☐ work ☐ study ☐ volunteering

Please name Job #2: ______________________________

5b. How many weeks in the <u>past 12 months</u> did you do Job # 2?	___ weeks
6b. How many days <u>per week</u> did you do Job # 2?	___ days
7b. How much time <u>per day</u> did you spend <u>sitting</u> for Job # 2?<br>*(include driving and travelling while doing this job; do not include time commuting to and from this job)*	___hr ___min

Think about the total time you spent sitting during Job # 2.

8b. How often did you "break up" the time you spent <u>sitting</u> in Job # 2?

☐	☐	☐	☐	☐
(less than hourly)	*(hourly)*	*(half hourly)*	*(every 10 mins)*	*(every 5 mins)*

OR ☐ I did not sit for more than 30 minutes in a day

➔ Did you have any other work, study or volunteering "jobs" in the past 12 months? If so, continue on the next page.

➔ If you did not have any other jobs, please skip to **Section 5** on page 11.

Choose type of “job”: ☐ work ☐ study ☐ volunteering

Please name Job #3 ______________________________

5c. How many weeks in the past 12 months did you do Job # 3? ___ weeks

6c. How many days per week did you do Job # 3? ___ days

7c. How much time per day did you spend sitting for Job # 3? ___hr ___min
(include driving and travelling while doing this job; do not include time commuting to and from this job)

Think about the total time you spent sitting during Job # 3.

8c. How often did you “break up” the time you spent sitting in Job # 3?

☐ *(less than hourly)* ☐ *(hourly)* ☐ *(half hourly)* ☐ *(every 10 mins)* ☐ *(every 5 mins)*

OR ☐ I did not sit for more than 30 minutes in a day

➔ Did you have any other work, study or volunteering “jobs” in the past 12 months? If so, continue on the next page.

➔ If you did not have any other jobs, please skip to **Section 5** on page 11.

Choose type of “job”: ☐ work ☐ study ☐ volunteering

Please name Job #4 ______________________________

5d. How many weeks in the past 12 months did you do Job # 4? ___ weeks

6d. How many days per week did you do Job # 4? ___ days

7d. How much time per day did you spend sitting for Job # 4? ___hr ___min
(include driving and travelling while doing this job; do not include time commuting to and from this job)

Think about the total time you spent sitting during Job # 4.

8d. How often did you “break up” the time you spent sitting in Job # 4?

☐	☐	☐	☐	☐
(less than hourly)	*(hourly)*	*(half hourly)*	*(every 10 mins)*	*(every 5 mins)*

OR ☐ I did not sit for more than 30 minutes in a day

SECTION 5 – CHILDCARE AND ELDER CARE

This section refers to the time you spent sitting while taking care of your children, grandchildren or elderly family members.

Were you involved in childcare or elder care each day, on either weekdays or weekends, over the past 12 months?

➔ If no, please write "0" in the response section, below.

Please record the usual amount of time you spent sitting during childcare or elder care over the past 12 months.

9. How long did you usually spend sitting or lying down while caring for your child per day? *(examples: nursing baby, helping child with homework)*	___hr ___min *(weekday)*	___hr ___min *(weekend)*
10. How long did you usually spend sitting down while caring for an elderly family member per day? *(examples: reading aloud, assistance with eating meals)*	___hr ___min *(weekday)*	___hr ___min *(weekend)*

SECTION 6 – LIGHT LEISURE AND RELAXING

This section refers to things done for enjoyment, during your own time.

Please record the usual amount of time you spent sitting or lying down in these pursuits over the past 12 months.

SCREEN TIME

11. How long did you usually spend watching TV (dvds/videos) or playing video games per day? ___hr ___min *(weekday)* ___hr ___min *(weekend)*

12. How long did you use a computer for leisure or for additional work on your own time per day? ___hr ___min *(weekday)* ___hr ___min *(weekend)*

Think about the total time you spent watching TV or using a computer during your leisure-time. We are interested in how often you stood up and moved around to "break up" the time you spent sitting or lying down. For example, you might have got up to get a cup of coffee during a commercial break.

13. How often did you "break up" the time you spent watching TV or using a computer during your leisure-time?

☐ *(less than hourly)* ☐ *(hourly)* ☐ *(half hourly)* ☐ *(every 10 mins)* ☐ *(every 5 mins)*

14. How often did you eat snack-foods (e.g. chips, sweets) while watching TV during your leisure-time?

☐ *(always)* ☐ *(usually)* ☐ *(sometimes)* ☐ *(rarely)* ☐ *(never)*

OTHER LEISURE PURSUITS

15. How long did you usually spend reading while sitting or lying down <u>per day</u>? ___hr ___min *(weekday)* ___hr ___min *(weekend)*

16. How long did you usually spend in other leisure pursuits while sitting down <u>per day</u>? ___hr ___min *(weekday)* ___hr ___min *(weekend)*

Some examples:

listening to music	*talking to friends*	*sewing/knitting*
doing crosswords/puzzles	*doing crafts*	*attending a sporting event*
woodworking	*playing cards*	*praying/meditating*
writing letter	*sitting outdoors*	*watching a movie at the cinema*

SECTION 7 – FINAL QUESTIONS

Were you involved in other daily pursuits done sitting or lying down that were not covered in this questionnaire, on either weekdays or weekends, over the past 12 months?

➔ If no, please continue to question 17.

Please record the usual amount of time you spent sitting or lying down in other pursuits not covered in this questionnaire.

Other pursuits

……………………………………..	___hr ___min *(weekday)*	___hr ___min *(weekend)*
……………………………………..	___hr ___min *(weekday)*	___hr ___min *(weekend)*
……………………………………..	___hr ___min *(weekday)*	___hr ___min *(weekend)*

17. Please estimate the amount of time it took to complete the SIT-Q ___hr ___min

18. Date questionnaire completed ___ / ___ / _____ *(dd)* *(mm)* *(year)*

Thank you for your help with this questionnaire.
